# Conservation and Variability in Mitochondrial Genomes of Perlodidae: Insights from Comparative Mitogenomics

**DOI:** 10.3390/insects16030245

**Published:** 2025-02-26

**Authors:** Xiao Yang, Qing-Bo Huo, Abdur Rehman, Ya-Fei Zhu, Yu-Zhou Du

**Affiliations:** 1 College of Plant Protection & Institute of Applied Entomology, Yangzhou University, Yangzhou 225009, China; 18883784145@163.com (X.Y.); jw30th@163.com (Q.-B.H.); rehman.ento@aup.edu.pk (A.R.); z1525747447@139.com (Y.-F.Z.); 2 Joint International Research Laboratory of Agriculture and Agri-Product Safety, the Ministry of Education, Yangzhou University, Yangzhou 225009, China

**Keywords:** mitochondrial, codon analyse, start codon, Plecoptera, Perlodidae

## Abstract

The mitochondrial genomes of three stoneflies, e.g., *Tibetisoperla wangluyui* Huo and Du, 2021, *Perlodinella kozlovi* Klapálek, 1912 and *Perlodinella epiproctalis* (Zwick, 1997), were sequenced in this study, with lengths 16,043 bp, 16,024 bp, and 16,071 bp, respectively. Each mitogenome contained 37 genes including 22 tRNAs, two ribosomal RNAs, 13 protein-coding genes (PCGs), and a noncoding control region (CR). In general, standard ATN start and TAN termination codons were evident in the PCGs. Meanwhile, in this paper, three newly published mitochondrial genomes and 11 existing mitochondrial genomes of the Perlodidae from NCBI were analyzed. Among the 13 PCGs in the mitochondrial genome of Perlodidae, the lengths of atp6, atp8, cox2, cox3, cytb, nad1, nad2, nad3, and nad4 are exactly the same, and the length of cox1 is 1536–1569 bp. The length of nad4L is 297, but the length of *Arcynopteryx dichroa* is 300. The length of nad5 ranges from 1732 bp to 1752 bp, while that of nad6 ranges from 525 bp to 534 bp. The length of rrnL is between 1292 and 391 bp, and the length of rrnS is between 793 and 869 bp. In addition, we found that atp8 in Isoperlinae started with GTG as a start codon but in Perlodinae, it started with ATG. Despite these advances, mitochondrial genome data from the Perlodidae are still needed.

## Simple Summary

The Perlidae family represents a major group within the order Plecoptera. Previously, very few mitochondrial genome sequences were available for this family (only 13 publicly accessible sequences exist to date). Compared with other families, the taxonomic coverage of Perlidae mitochondrial genomes remains incomplete. Existing studies have merely provided phylogenetic reconstruction outcomes without conducting specific analyses or discussions on the mitochondrial genome architecture of this family. Therefore, this study aims to advance our understanding of mitochondrial genome organization in Perlidae, offering a more comprehensive perspective.

## 1. Introduction

The stonefly is a group of hemimetabolous aquatic insects that includes over 4500 valid species [1,2]. Stoneflies undergo hemimetabolous development, featuring egg, nymph, and adult stages. Nymphs require high levels of dissolved oxygen and, thus, prefer cool to cold, fast-flowing riffle areas of streams where coarse mineral substrates predominate [3]. In addition, the stonefly is also part of the treasure fish bait [4]. It is also sensitive to water quality and is an important insect for water quality detection, alongside mayflies and caddisflies [5,6]. Recently, the molecular biology of stoneflies has become a research focus due to their important economic and ecological value [7,8,9,10,11,12,13,14,15].

The order Plecoptera comprises 17 extant families, categorized into two suborders: Arctoperlaria and Antarctoperlaria [2]. The infraorder Systellognatha is a well-supported monophyletic group within Arctoperlaria, containing seven families across two superfamilies: Perloidea and Pteronarcyoidea. Perloidea includes the families Chloroperlidae, Perlidae, and Perlodidae, while Pteronarcyoidea consists of Peltoperlidae, Pteronarcyidae, and Styloperlidae [1]. Perlodidae consists of over 250 described species across 55 genera, with a distribution primarily in the Nearctic, Palaearctic, and Oriental regions [2].

The study of mitochondrial genomes in the order Plecoptera can be traced back to the analysis of Pteronarcys princeps (Pteronarcyidae) by Stewart and Beckenbach [16]. Over the past 15 years, a significant portion of sequencing and analysis has been conducted primarily by Chinese scholars [17,18,19,20,21,22,23,24,25,26,27,28,29].

These studies have covered most families within the two superfamilies of the suborder Arctoperlaria, including the families Perlidae, Perlodidae, Chloroperlidae, Peltoperlidae, Taeniopterygidae, and others. Protein-coding genes (PCGs) or combinations of PCGs and RNA genes have been widely utilized in the phylogenetic reconstruction of various stonefly groups. In the majority of these studies, the structure of the mitochondrial genomes has been described in detail and compared with other members of the class Insecta and even the entire Metazoa. Currently, the mitochondrial genomes of stoneflies typically contain the insect-typical 13 PCGs, 22 tRNA-coding genes, and 2 rRNA-coding genes, which are conserved in most insect mitochondrial genomes and are believed to be identical to the ancestral gene sequences of insects. The AT content varies approximately between 60% and 70% among different groups, with variations observed across taxa. Most PCGs use the standard ATN as start codons and TAN as stop codons. All 21 tRNAs exhibit the typical cloverleaf secondary structure, although some genes lack stem-loop structures in their arms. Stem-loop (SL) structures and tandem repeat sequences have been identified in the control region (CR). Additionally, these mitochondrial genomes have been extensively used for phylogenetic reconstruction, although some studies have only provided simple phylogenetic trees without detailed descriptions or analyses of their structural composition [24,25,26,27].

The family Perlodidae is a major group within the superfamily Perloidea of the suborder Arctoperlaria. Previously, very few mitochondrial genome sequences have been available for this family (currently, there are only 13 publicly available sequences). Compared to other families, the taxonomic coverage of mitochondrial genomes in Perlodidae is incomplete. Studies have only provided the results of phylogenetic reconstruction, without any specific analysis or discussion of the mitochondrial genome structure of this family [24,26]. Therefore, this paper aims to provide further insights into the mitochondrial structure of Perlodidae, offering a more comprehensive perspective.

## 2. Material and Methods

### 2.1. Sample Preparation and DNA Extraction

This study was conducted without harming protected or endangered species and all research activities were authorized. *Tibetisoperla wangluyui* was collected from Tibet, China in May 2021, *Perlodinella kozlovi* was collected from Qinghai Province, China in July 2020, and *Perlodinella epiproctalis* was collected from Shaanxi Province, China in May 2020; all specimens were preserved in 100% ethanol and stored at −20 °C. Genomic DNA was extracted from the legs of specimens with the Column mtDNAout Kit (Axygen Biotechnology, Hangzhou, China) as recommended by the manufacturer and stored at −20 °C until used for PCR.

### 2.2. PCR Amplification and Sequencing

Mitochondrial genome was amplified using LA–PCR and continuous specific PCR amplification with the following conditions: Perform initial denaturation at 93 °C for 2 min, and then perform 40 cycles at 92 °C for 10 s; annealing at 54 °C for 30 s; and stretching at 68 °C (20 cycles) for 8 min Elongation rate, which increases by 20 s/cycle in the last 20 cycles. The final extension is 10 min at 68 °C. PCR products were purified with Axygen DNA Gel Extraction Kit (Axygen Biotechnology, Hangzhou, China), and quality control was subsequently carried out on the purified DNA samples. The quality of DNA was assessed using qubit3.0 and 1% agarose gel electrophoresis.

High-qualified DNA samples were applied to 500 bp paired-end library construction using the NEBNext Ultra DNA Library Prep Kit for Illumina sequencing. Sequencing was carried out on the Illumina NovaSeq 6000 platform (BIOZERON Co., Ltd., Shanghai, China). De novo assembly with GetOrganelle v1.6.4 referencing mitogenome of closely related species produced contigs of mitogenome. A number of potential mitochondrion reads were extracted from the pool of Illumina reads using BLAST searches against mitogenomes of related species and the GetOrganelle v1.6.4 results. The mitochondrion Illumina reads were obtained to perform complete mitogenome de novo assembly using the SPAdes–3.13.1 package. The GetOrganelle assembly contig was optimized by the scaffolds from SPAdes 3.13.0 result. Finally, the assembled sequences were reordered and oriented according to the reference mitogenome, thus generating the final assembled mitochondrion genomic sequence (BIOZERON Co., Ltd., Shanghai, China).

### 2.3. Mitogenome Assembly and Annotation

The mitogenome assembly was performed using CodonCode Aligner (https://www.codoncode.com/aligner/, accessed on 5 February 2023). The mitogenomes from other species of order Plecoptera were used to recognize rRNAs, tRNAs, and PCGs genes, and ORFs were enclosed through ORF finder (https://www.ncbi.nlm.nih.gov/orffinder/, accessed on 5 February 2022). The CGview tool server was used to create a circular map of the mitogenome [30]. The structure of tRNA was predicted by the ARWEN algorithm using its default settings [31]. Sequence comparisons were conducted in PhyloSuite software (https://dongzhang0725.github.io/, accessed on 5 February 2023) to evaluate nucleotide composition and relative synonymous codon usage (RSCU) among the Perlodidae mitogenomes [32]. AT and GC skew analyses were calculated using the formulas AT − skew = (A − T)/(A + T) and GC − skew = (G − C)/(G + C) [33]. Mitogenome sequences of this research were deposited in the GenBank (Table 1).

## 3. Results and Discussion

### 3.1. Mitogenome Organization and Base Composition

**Figure 1:**
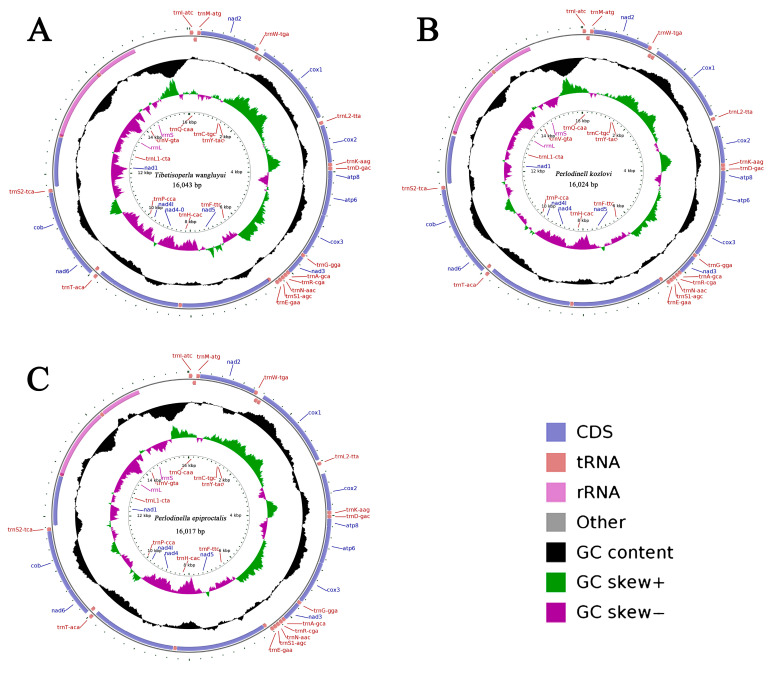
Map of the newly sequenced mitogenome. (**A**): *Tibetisoperla wangluyui*; (**B**): *Perlodinella kozlovi*; (**C**): *Perlodinella epiproctalis*. The interior spheres showed GC content (guanine and cytosine nucleotides) and the GC skew is plotted as the deviation from the overall mean value of the whole sequence.

The complete mitogenomes of *T. wangluyui*, *P. kozlovi* and *P. epiproctalis* are 16,043 bp, 16,024 bp, and 16,071 bp in size, respectively (Figure 1, Table 1). The length of completely sequenced mitogenomes was medium-sized, about 16,000 bp, when compared with the mitogenomes of other Perlodidae species (Table 1). The mitogenome is a circular DNA molecule containing 13 PCGs, 22 tRNAs, 2 rRNA genes, and a control region (Tables S1–S3). The PCG, tRNA, rRNA and non-coding region each part of the length is not much different. The three newly published mitochondrial genomes found no genetic rearrangement, which was the same as the ancestral gene order of *Drosophila yakuba* [34].

In *T. wangluyui*, *P. kozlovi*, and *P. epiproctalis*, the A + T content was as follows: 67.1%, 69.4%, and 70.2% (whole mitogenomes); 64.9%, 68%, and 69.2% (PCGs); 68.5%, 68.7%, and 68.9% (tRNAs); and 70.9%, 70.8%, and 71.2% (rRNAs); respectively (Table 2). In the PCGs of *Tibetisoperla wangluyui*, the highest and lowest A + T content was 73.1% for nad4L and 60.1% for cox1. In the PCGs of *P. kozlovi*, the highest and lowest A + T content was 76.1% for nad4L and 63% for cox1. In the PCGs of *P. epiproctalis*, the highest and lowest A + T content was 76.8% for nad4L and 64.5% for cox1. The A + T contents of whole mitogenomes, PCGs, tRNAs, and rRNAs in *P. epiproctalis* were all the highest. In view of this phenomenon, it is necessary to collect more specimens from different environments and extract more molecular data for more accurate exploration.

### 3.2. Protein-Coding Genes

The 13 PCGs of the three Perlodidae mitogenomes were similar in size and A + T content (Tables S1–S3). The majority of the PCGs in all three mitogenomes initiated with the standard start codon ATN (ATT, ATC, ATA, and ATG). However, nad1 in *T. wangluyui* started with TTG and atp8 and nad5 used GTG as a start codon. In *P. kozlovi* and *P. epiproctalis*, nad1 started with TTG (Tables S4–S6). Most PCGs had standard stop codons (TAA or TAG); however, cox2 in both *P. kozlovi* and *P. epiproctalis* and cox1, cox2 in *T. wangluyui* contained a truncated termination codon (‘T’). Some PCG genes used nonstandard start codons or stop codons, these phenomena are common in Plecoptera [28,29].

**Figure 2:**
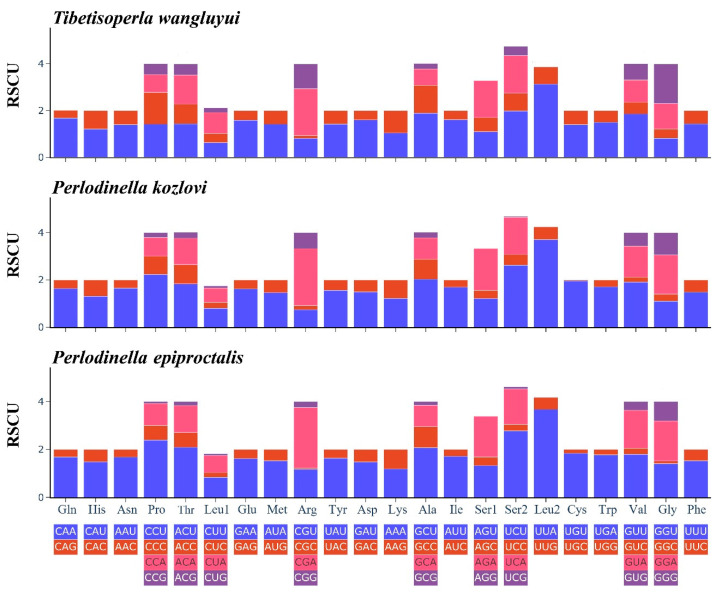
The relative synonymous codon usage (RSCU) in the *Tibetisoperla wangluyui*, *Perlodinella kozlovi*, and *Perlodinella epiproctalis* mitogenomes. The X-axis shows different amino acids, and the Y-axis shows the RSCU value (the number of times a certain synonymous codon is used/the average number of times that all codons coding the amino acid are used).

We calculated the RSCU values for the mitogenomes of our newly sequenced species (Figure 2). In *T. wangluyui*, *P. kozlovi*, and *P. epiproctalis*, UUA (Leu2), UUU (Phe), and AUU (Ile) were relatively high, whereas CGC (Arg) was used the least (Figure 2).

### 3.3. Transfer RNA Genes

**Figure 3:**
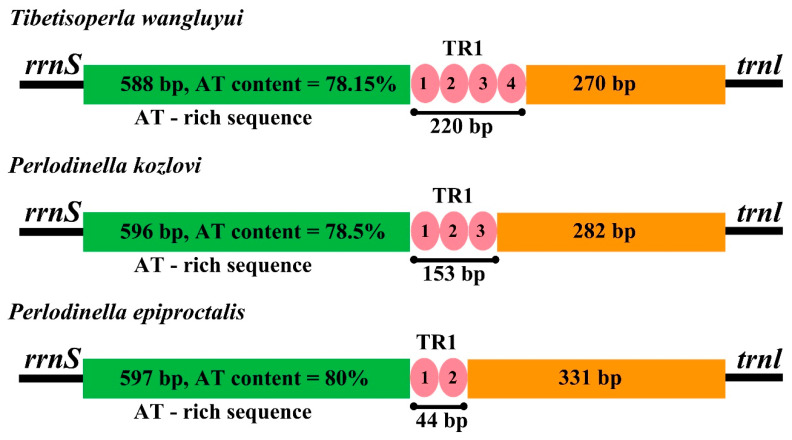
The structural elements in the control region of the family Perlodidae mitogenomes. The CR is divided into three regions: the leading sequence adjacent to srRNA with high AT content (green), tandem repeated sequence blocks (pink), and the remainder of the control region (orange).

The mitogenome of Perlodidae contains 22 scattered transfer RNA (tRNA) genes interspersed with protein-coding genes (PCGs) and ribosomal RNA (rRNA) genes (Tables S4–S6). The lengths of *T. wangluyui*, *P. kozlovi*, and *P. epiproctalis* tRNA genes were 1478 bp, 1479 bp, and 1479 bp, and the A + T content of tRNA genes was 68.5%, 68.5%, and 68.9%, respectively (Tables S1–S3). Individual tRNA lengths range from 64 bp to 71 bp across species (Figures S1–S3. Most tRNAs had a typical cloverleaf secondary structure (Figure 3 and Figures S1–S3). The anticodons of the 22 tRNAs in the three Perlodidae species were identical to those of other stoneflies. The tRNAs contained mismatched base pairs, and most of these were G–U pairs (Figures S1–S3).

### 3.4. Ribosomal RNA Genes

There were two rRNAs in the Perlodidae mitogenomes. The length of each species, *T. wangluyui* (2180 bp), *P. kozlovi* (2153 bp), and *P. epiproctalis* (2183 bp), had an A + T content that ranged from 70.8% to 71.2% (Tables S1–S3). The *rrnL* genes were 1385 bp with an A + T content of 72.4% in *T. wangluyui*, 1355 bp with an A + T content of 73.1% in *P. kozlovi*, and 1384 bp with an A + T content of 73.3% in *P. epiproctalis*. Meanwhile, the small ribosomal RNA gene (*rrnS*) was 795 bp with an A+T content of 65.9% in *T. wangluyui*, 798 bp with an A + T content of 67.04% in *P. kozlovi*, and 799 bp with an A + T content of 67.6% in *P. epiproctalis* (Tables S3–S6). Both *lrRNA* and *srRNA* size and characteristics are consistent with those reported in well-documented Plecoptera species [35].

### 3.5. The Control Region

This control region is crucial for initiating transcription and replication processes [36], making its variability significant for understanding mitochondrial function and evolutionary relationships. The CR lengths for the species studied are as follows: *T. wangluyui*, 1115 bp; *P. kozlovi*, 1061 bp; and *P. epiproctalis*, 1007 bp, with A + T content of 78.15%, 78.5%, 80%, respectively, all located between the *rrnS* and *trnLle* genes (Figure 2, Table 2). The CR lengths in the three species mitogenomes are comparable to those found in other stoneflies, which also exhibit high A + T content. The CR of the Perlodidae mitogenomes can be divided into three distinct regions: (1) a leading sequence adjacent to the *srRNA* with a high A + T content, (2) a tandem repeat (TR) sequence block comprising multiple repeat units, and (3) the remaining portion of the control region (Figure 3).

### 3.6. Mitochondrial Genome Analysis of Perlodidae

In this paper, three newly sequenced mitochondrial genomes of the Perlodidae and 11 existing mitochondrial genomes of the Perlodidae from NCBI were analyzed. Among the 13 PCGs in the mitochondrial genome of Perlodidae, the lengths of atp6, atp8, cox2, cox3, cytb, nad1, nad2, nad3, and nad4 are exactly the same, and the length of cox1 is 1536–1569 bp. The length of nad4L is 297, the length of *Arcynopteryx dichroa* is 300, the length of nad5 is between 1732 and 1752 bp, and the length of nad6 is between 525 and 534 bp. The length of rrnL is between 1292 and 1391 bp, and the length of rrnS is between 793 and 869 bp (Table S7).

The majority of the PCGs in all three mitogenomes initiated with the standard start codon ATN (ATT, ATC, ATA, and ATG). However, atp6 in *Megarcys ochracea* started with GTG as a start codon; atp8 in Isoperlinae started with GTG as a start codon; cox1 in *Filchneria zhouchangfai* started with CGA as a start codon; nad1 in Perlodidae started with TTG as a start codon; and nad5 in *Megarcys ochracea*, *Skwala compacta*, *Arcynopteryx dichroa*, and Isoperlinae started with GTG as a start codon (Table S7).

Most PCGs had standard stop codons (TAA or TAG). However, cox1 in *Arcynopteryx dichroa*, *T. wangluyui*, and *Isoperla qinlinga* contained a truncated termination codon (‘T’); nad2 in *Arcynopteryx dichroa* contained a truncated termination codon (‘T’); nad5 contained three difference putative terminal codon (‘T’, ‘TA’, and ‘TAA’); and cox2 in Perlododae contained a truncated termination codon (‘T’) (Table S7).

From the point of view of AT content, Perlodinae were generally higher than Isoperlinae in the whole mitochondrial genome, especially in PCGs, but not in tRNA and rRNA.

## Figures and Tables

**Table 1:** Mitochondrial genome used in this study.

Family	Organism	Number	AT%	GenBank Number
Perlodidae	*Isoperla bilineata*	15,048	67.8	NC_038190.1
	*Isoperla eximia*	16,034	67.8	NC_038167.1
	*Arcynopteryx dichroa*	16,215	69.3	NC_059845.1
	*Filchneria songi*	16,028	70.1	MZ475123.1
	*Filchneria zhouchangfai*	16,032	69.8	NC_086967.1
	*Perlodes* sp.	16,039	70.4	MF197377.1
	*Perlodinella shennongjia*	17,612	70.7	NC_086966.1
	*Pseudomegarcys japonica*	16,067	66.5	NC_038168.1
	*Stavsolus manchuricus*	16,130	66.7	PQ616052
	*Neowuia wuyishana*	16,672	70	PQ616053
	*Perlodinella kozlovi*	16,024	69.4	PQ616054
	*Perlodinella epiproctalis*	16,017	70.2	PQ616056
	*Isoperla qinlinga*	16,195	68.5	PQ616055
	*Tibetisoperla wangluyui*	16,043	67.1	PQ644302
	*Megarcys ochracea*	16,239	67.4	PQ222379
	*Skwala compacta*	16,418	69.2	PP997962
Leuctridae	*Rhopalopsole subnigra*	15,562	69.7	OQ612622.1
	*Rhopalopsole bulbifera*	15,599	70.7	NC_042207.1

**Table 2:** The nucleotide components of mitochondrial genomes of Perlodidae.

Subfamily	Species	Length (bp)	GC Skew	A (%)	T (%)	C (%)	G (%)	A + T (%)	AT Skew (+)	AT Skew (−)
Perlodinae	*Neowuia wuyishana*	16,672	−0.241	36.2	33.8	18.6	11.3	70	0.034	−0.034
	*Megarcys ochracea*	16,239	0.266	31.9	35.5	11.9	20.7	67.4	−0.053	0.053
	*Stavsolus manchuricus*	16,130	−0.248	34.6	32.1	20.8	12.5	66.7	0.039	−0.039
	*Skwala compacta*	16,418	0.238	32.6	36.6	11.7	19.1	69.2	−0.057	0.057
	*Pseudomegarcys japonica*	16,067	−0.279	35.4	31.1	21.5	12	66.5	0.066	−0.066
	*Perlodinella shennongjia*	17,612	−0.212	37.1	33.6	17.9	11.4	70.7	0.049	−0.049
	*Perlodinella kozlovi*	16,024	−0.218	35.9	33.5	18.7	12	69.4	0.035	−0.035
	*Perlodinella epiproctalis*	16,017	−0.214	36.2	34	18.1	11.7	70.2	0.031	−0.031
	*Filchneria zhouchangfai*	16,032	−0.228	36.5	33.3	18.5	11.7	69.8	0.047	−0.047
	*Filchneria songi*	16,028	−0.199	36.5	33.6	18	12	70.1	0.04	−0.04
	*Arcynopteryx dichroa*	16,215	−0.238	36.5	32.8	19	11.7	69.3	0.053	−0.053
Isoperlinae	*Tibetisoperla wangluyui*	16,043	−0.233	34.7	32.4	20.3	12.6	67.1	0.035	−0.035
	*Isoperla qinlinga*	16,195	−0.234	35.3	33.2	19.4	12.1	68.5	0.031	−0.031
	*Isoperla eximia*	16,034	−0.226	35	32.8	19.8	12.4	67.8	0.031	−0.031
	*Isoperla bilineata*	15,048	−0.229	34.6	33.2	19.8	12.4	67.8	0.022	−0.022

## Data Availability

The data that support the findings of this study are openly available in NCBI: GenBank accession nos. of mitochondrial gene: PQ644302, PQ616054 and PQ616056.

## Supplementary Materials

The following supporting information can be downloaded at: https://www.mdpi.com/article/10.3390/insects16030245/s1, Table S1: The nucleotide components of the *Tibetisoperla wangluyui* mitogenomes; Table S2: The nucleotide components of the *Perlodinella kozlovi* mitogenomes; Table S3: The nucleotide components of the *Perlodinella epiproctalis* mitogenomes; Table S4: Organization of the *Tibetisoperla wangluyui* mitochondrial genome.; Table S5: Organization of the *Perlodinella kozlovi* mitochondrial genome; Table S6: Organization of the *Perlodinella epiproctalis* mitochondrial genome; Table S7: Organization of the Perlodidae mitochondrial genome; Figure S1: Secondary structures of 22 tRNAs of *Tibetisoperla wangluyui*; Figure S2: Secondary structures of 22 tRNAs of *Perlodinella kozlovi*; Figure S3: Secondary structures of 22 tRNAs of *Perlodinella epiproctalis*.
